# The Role of PKM2 in the Regulation of Mitochondrial Function: Focus on Mitochondrial Metabolism, Oxidative Stress, Dynamic, and Apoptosis. PKM2 in Mitochondrial Function

**DOI:** 10.1155/2022/7702681

**Published:** 2022-05-06

**Authors:** Jing Gao, Yuwei Zhao, Tao Li, Xueqi Gan, Haiyang Yu

**Affiliations:** ^1^State Key Laboratory of Oral Diseases, National Clinical Research Center for Oral Diseases, West China Hospital of Stomatology, Sichuan University, Chengdu 610041, China; ^2^Laboratory of Mitochondrial and Metabolism, Department of Anesthesiology, National Clinical Research Center for Geriatrics, West China Hospital of Sichuan University, Chengdu 610041, China

## Abstract

The M2 isoform of pyruvate kinase (PKM2) is one isoform of pyruvate kinase (PK). PKM2 is expressed at high levels during embryonic development and tumor progression and is subject to complex allosteric regulation. PKM2 is a special glycolytic enzyme that regulates the final step of glycolysis; the role of PKM2 in the metabolism, survival, and apoptosis of cancer cells has received increasing attention. Mitochondria are directly or indirectly involved in the regulation of energy metabolism, susceptibility to oxidative stress, and cell death; however, the role of PKM2 in mitochondrial functions remains unclear. Herein, we review the related mechanisms of the role of PKM2 in the regulation of mitochondrial functions from the aspects of metabolism, reactive oxygen species (ROS), dynamic, and apoptosis, which can be highlighted as a target for the clinical management of cardiovascular and metabolic diseases.

## 1. Introduction

Pyruvate kinase (PK) is one of the key enzymes of glycolysis. PK can catalyze the transphosphorylation from phosphoenolpyruvate (PEP) to ADP as the last step of glycolysis to generate ATP [1]. The M2 isoform of pyruvate kinase (PKM2) is distributed in tissues such as the brain, liver, and tumor tissues [2, 3]. PKM2 has two different forms: a dimeric form and a tetrameric form [4]. The tetrameric form of PKM2 has a higher PK enzymatic activity to catalyze the production of pyruvate [2]. The dimer form of PKM2 has a low PK activity and is closely related to biosyntheses such as energy metabolism and material synthesis [5]. PKM2 expression is associated with several biological activities, including regulation of tumor growth [6], embryogenesis [7], tissue regeneration [8], and inflammatory regulation [9]. Some scholars have suggested that PKM2 can attach to the mitochondrial outer membrane to maintain mitochondrial function; the involvement of PKM2 in the regulation of mitochondrial functions has received increasing attention [10–12].

Mitochondria are important organelles that play a pivotal role in cell life and cell death. Mitochondria are dynamic organelles that consistently migrate, fuse, and divide to modulate their number, size, and shape [13]. In addition, mitochondria play a crucial role in different physiological processes, especially in energy production, the generation of reactive oxygen species, and calcium signaling [14]. Thus, mitochondrial dysfunction leads to various diseases, such as metabolic diseases and cancer [15]. Accumulating evidence suggests that PKM2 may participate in the regulation of the mitochondrial physiological process [10–12]. Therefore, this review focuses on the role of PKM2 in mitochondrial physiological function with respect to metabolism, oxidative stress, dynamic, and apoptosis.

## 2. PKM2 Expression and Its Biological Functions

There are four tissue-specific isoforms of PK in mammals, including PKM1, PKM2, PKL, and PKR [16]. PKL and PKR are predominantly expressed in the liver and erythrocytes, respectively. PKM1 is abundantly expressed in high-energy demanding organs such as the heart, brain, and muscle, while PKM2 is highly expressed in various proliferating cells, especially embryonic and tumor tissues [3]. PKM1 and PKM2 are produced through alternative splicing under the regulation of several splicing factors, such as heterogeneous nuclear ribonucleoproteins (hnRNPs) and polypyrimidine-tract binding (PTB) [17]. PTBP1 leads to the expression of PKM2 through blocking the inclusion of exon 9 and inducing the inclusion of exon 10 [18].

PKM2, existing as tetrameric and dimeric forms, has been found in the nucleus, mitochondria, and extracellular secretion [19]. The dimeric PKM2 can enter the nucleus, and PKM2 in the nucleus functions as a co-activator of the transcription factor to activate the transcription of target genes that are involved in mitochondrial biogenesis [20]. The subcellular PKM2 can be regulated by multiple signaling pathways, including the phosphorylation of PKM2 at tyrosine, serine, and threonine residues [21], acetylation of PKM2 at K305 [3] succinylation [11], and O-GlcNAcylation [22].

PKM2 exerts several biological functions [20]. PKM2 is highly expressed and shifts the glucose metabolism from mitochondrial respiration to lactate production in tumor cells; therefore, PKM2 may serve as a potential diagnostic marker in cancer [23]. The inhibitors and activators of PKM2 can be promising anti-cancer drugs. Exerting a similar role, PKM2 may also act as a key protein kinase in other diseases [9, 17, 24]. PKM2 is a requisite for Th1 and Th17 differentiation and may be a therapeutic target for T cell–dependent autoimmune diseases [25]. PKM2 is also involved in renal inflammation in type 2 diabetic nephropathy by promoting the phosphorylation of STAT3 and NF-*κ*B [24].

## 3. Mitochondrial Function

Mitochondria are essential components of eukaryotic life [26]. Mitochondria are comprised of two separate and functionally distinct outer membranes (OMs) and inner membranes (IMs), that encapsulate the intermembrane space (IMS) and matrix compartments [27]. Mitochondria contain a circular genome, mitochondrial DNA (mtDNA), which qualify mitochondria for the function of semi-conservative replication, transcription, and translation [28].

Mitochondria are the energy-producing organelles of the cell; they can generate the majority of a cell's ATP via oxidative phosphorylation (OXPHOS) [29]. Glucose is metabolized to ATP for energy supply via two types of reactions, OXPHOS in mitochondria and aerobic glycolysis in the cytosol [30, 31]. Mitochondria are the major intracellular source of reactive oxygen species (ROS); mitochondrial ROS originates from respiratory chain complexes, particularly at the level of complex III and complex I [32]. ROS can cause cumulative damage to mitochondria and mtDNA, leading to mitochondrial dysfunction, which further causes ROS production and mtDNA damage [33].

Mitochondria are dynamic organelles that undergo a dynamic cycle of transport, fission, and fusion. The mitochondrial dynamics maintain the shape, distribution, and size of mitochondria [34]. In mammals, mitochondrial fusion is mediated by mitofusion (Mfn1 and Mfn2, located in the OMs) and optic atrophy gene 1 (Opa1, located in the IMs) [35]. Meanwhile, mitochondrial fission is mediated by fission 1 protein (Fis1, located in the OMs) and dynamin-related protein 1 (Drp1, which is mostly cytosolic and translocates to the OMs during fission) [36]. Imbalanced mitochondrial dynamics lead to mitochondrial dysfunction.

Mitochondria exert multiple functions in the life process, including the control of stress responses, cell signal regulation, and cell apoptosis [37]. The molecular mechanisms underlying function regulation of mitochondria remain elusive, but numerous investigations have documented that PKM2 may be involved in the regulation of the mitochondrial functions.

## 4. The Effect of PKM2 on Mitochondrial Function

### 4.1. PKM2 and the Regulation of Mitochondrial Metabolism

PKM2 favors aerobic glycolysis, where glucose is primarily catabolized to lactate, rather than fully metabolized to carbon dioxide using mitochondrial OXPHOS [38]. This phenomenon is termed as the Warburg effect [39, 40].

The function of PKM2 as a metabolic switch can be regulated via three pathways. First, the expression of PKM2 is closely associated with cell metabolism [41, 42]; increased PKM2/PKM1 ratio has been reported to promote aerobic glycolysis. Several factors, such as never in mitosis (NIMA)-related kinase 2 (NEK2) [43], Sam68 [44], Fenofibrate [45], and SNHG6 [46], have been proven to affect glycolysis pathway by regulating the proportion of PKM2/PKM1. Second, the forms of PKM2 can also affect cell metabolism. Tetrameric PKM2 exhibits high catalytic activity to catalyze the production of pyruvate by PEP, promoting the flux of glucose-derived carbons to OXPHOS [19], while dimeric PKM2 is the less active state of PKM2 that facilitates the glycolytic intermediates for aerobic glycolysis pathways [47]. Third, some factors can also affect the glycolysis pathway by regulating the transport of PKM2 mRNA. T cells upregulate PKM2 expression through the mTOR1-HIF1 signaling [48], and the nuclear translocation of dimeric PKM2 is improved to increase the STAT3 phosphorylation in T cells [49], which further enhances Th1 and Th17 differentiation by promoting the glycolysis metabolism, representing as a therapeutic target for T cell–dependent autoimmune diseases [25].

**Figure 1:**
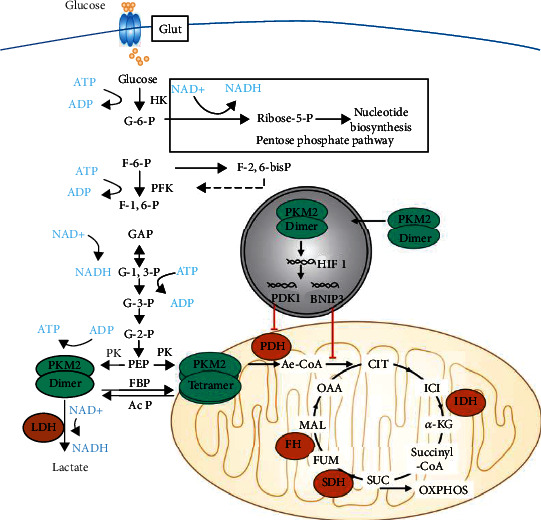
PKM2 promotes aerobic glycolysis and shifts the glucose metabolism from mitochondrial OXPHOS to aerobic glycolysis. PKM2 can activate the transcriptions of HIF-1, subsequently PDK1 and BNIP3. PDK1 inhibits the mitochondrial PDH to inhibit the conversion of pyruvate to acetyl-CoA. BNIP3 reduces the levels of mitochondrial-encoded proteins involved in OXPHOS. PKM2 induces the phosphorylation of AMP-activated protein kinase (AMPK), and thus phosphorylates and inhibits ACC.

With insights into the mechanisms underlying the effect of PKM2 on mitochondrial metabolism, several signaling pathways are involved [50]. First, PKM2 leads to a reduction of TCA intermediates. PKM2 can activate the transcriptions of HIF-1 and subsequently pyruvate dehydrogenase kinase 1 (PDK1) [51], and Bcl2-interacting protein 3 (BNIP3) [52]. PDK1 inhibits the mitochondrial PDH to inhibit the conversion of pyruvate to acetyl-CoA [53]. BNIP3 reduces the levels of mitochondrial-encoded proteins involved in OXPHOS [52]. Second, PKM2 reduces mitochondrial activity without damaging the mitochondria. PKM2 induces the phosphorylation of AMP-activated protein kinase (AMPK), a known inducer of mitochondrial activity [54]. Collectively, PKM2 shifts the glucose metabolism from mitochondrial OXPHOS to aerobic glycolysis, thereby acting as a potential diagnostic marker for tumors (Figure 1).

### 4.2. PKM2 and the Regulation of Mitochondrial ROS Signaling

PKM2 expression and the PKM2/PKM1 ratio are associated with the production of ROS [38–40]. However, no agreement has been reached about the positive or negative effect of PKM2 on ROS production. Some studies have reported that higher PKM2 expression leads to the decreased ROS production. PKM2 activation by TEPP-46 can decrease ROS production induced by either high-glucose [41] or inflammasome activation [42]. Another activator of PKM2, DASA-58, has also been proven to maintain ROS at a low level [43]. Besides, inhibition of the expression level of PKM2 by the PKM2-siRNA interference significantly stimulated ROS overproduction [44]. In contrast, some other studies came to the opposite conclusions [45–48]. Shuvalov et al. [46] have reported that the overexpression of PKM2 causes elevation of the membrane mitochondrial potential (MMP), subsequently leading to an increase in ROS production. Song et al. [47] have also proven that PCB126-increased ROS production is associated with activation of PKM2/STAT3/Snail1 cascades. It has been reported that PKM2 knockdown effectively relieved the increased ROS level in pancreatic cancer [48].

While PKM2 is involved in ROS regulation, several studies have proven that ROS exerts a negative effect on PKM2 [49–53]. The ROS inhibition of PKM2 may be involved in several signaling. ROS can oxidize PKM2 Cys358, further decrease the active tetramer and promote the phosphorylation of PKM2, thereby causing the inhibition of PKM2 activity [44, 54]. Xiangyun et al. [55] have also reported that high concentrations of ROS can decrease both the succinylation and activity of PKM2 by increasing its binding to SIRT5. Furthermore, ROS can not only affect the expression level and localization of PKM2 but also mediate the crosstalk between PKM2 and other enzymes, including apurinic/apyrimidinic endonuclease (APE1) and ectonucleotide pyrophosphatase/phosphodiesterase 2 (ENPP2) [56, 57]. While another study has shown that the PKM2 mRNA is not inhibited by ROS, the decrease of PKM2 expression is caused by PKM2 degradation. The interaction of PKM2 and ROS can also increase the cell sensitivity to ROS [58]. The increased level of ROS induces mitochondrial translocation of PKM2, and mitochondrial PKM2 interacts with and phosphorylates Bcl2, which inhibits ROS-induced apoptosis [10]. Besides, ROS-dependent inhibition of PKM2 may promote glucose influx into the pentose phosphate pathway (PPP), which contributes to a metabolic response that can deplete ROS [59].

### 4.3. PKM2 and Mitochondrial Dynamic

**Figure 2:**
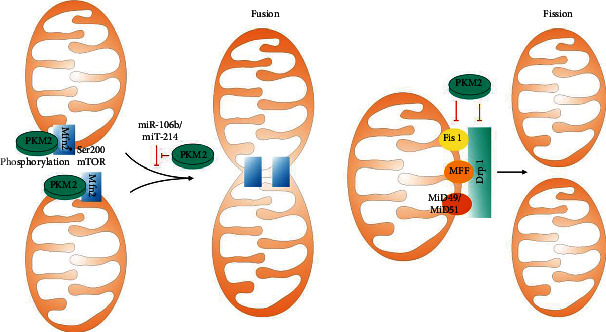
PKM2 regulates mitochondrial dynamics, including decreasing the numbers and increasing the sizes of mitochondria. PKM2 can translocate to mitochondria and inhibit mitochondria fission by downregulating the expression of Drp1. PKM2 also inhibits the expression of Fis1. PKM2 promotes mitochondrial fusion by triggering the Mfn2 expression. The interaction of PKM2 and Mfn2 can be increased by mTOR-mediated phosphorylation of Mfn2 at Ser200, and smiR-106b is associated with the down-regulation of Mfn2 expression and the PKM2 mediation of mitochondrial fusion. Besides, miR-214 targets Mfn2 by impairing its binding with PKM2.

Overexpression of PKM2 can regulate mitochondrial dynamics, including decreasing the numbers and increasing the sizes of mitochondria [41] (Figure 2). PKM2 can translocate to mitochondria and inhibit mitochondria fission by downregulating the expression of Drp1 [60]. Drp1 activity relates to its binding partners on the OMs, including mitochondrial fission factor (MFF), Fis1, and mitochondrial dynamics proteins of 49 and 51 kDa (MiD49 and MiD51). PKM2 can inhibit the expression of Fis1 [43], and future studies can be conducted to explore the relationship of PKM2 with MFF, MiD51, and MiD49.

PKM2 promotes mitochondrial fusion by triggering the Mfn2 expression [61, 62]. The interaction of PKM2 and Mfn2 can be increased by mammalian target of rapamycin (mTOR)-mediated phosphorylation of Mfn2 at Ser200 [12]. Several microRNAs are involved in this process; smiR-106b is associated with the down-regulation of Mfn2 expression and the PKM2 mediation of mitochondrial fusion [63]. Besides, miR-214 targets Mfn2 by impairing its binding with PKM2 [64]. Others like mitochondrial MiD51 [65] and mitochondrial calcium uniporter complex (MCUC) [66] may be the potential mechanism linking PKM2 and mitochondrial dynamics.

The effect of PKM2 on mitochondrial autophagy still requires validation with additional experiments. On the one hand, PKM2 contributes to mitochondrial autophagy via the HIF-1/BNIP3 pathway in hypoxic and some cancer cells [67, 68]. PKM2 activates the transcription of BNIP3 by inducing HIF-1*α*; BNIP3 can induce mitochondrial membrane permeabilization via Bax/Bak1 or via the opening of the mPTP, which leads to release of mitochondrial pro-death proteins and activation of cell death [67, 69]. On the other hand, PKM2 inhibits autophagy by activating the mTOR [70, 71]. PKM2 activates mTORC1 via the PI3K-Akt signaling pathways to inhibit autophagy in Hela, HEK293T, and HCT116 cells [71]. PKM2 overexpression may phosphorylate S202/203 of AKT1S1 and their phosphorylation activates mTORC1 [72]. Besides, PKM2 may reduce the ratio of AMP/ATP and the inhibition of AMP to mTORC1 kinase activity and autophagy in A549 cells [73].

### 4.4. PKM2 and Mitochondrial Apoptosis Pathway

**Figure 3:**
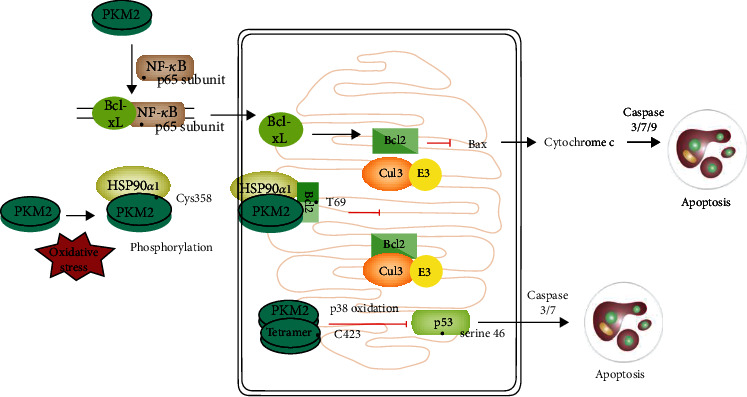
The metabolic function of PKM2 is involved in modulating mitochondrial apoptosis of cancer cells; the enhanced glycolysis by PKM2 can attenuate cell apoptosis in cancer cells. PKM2 translocates to the outer membrane of mitochondria under oxidative stress. In the mitochondria, tetrameric PKM2 suppresses the p53 transcriptional activity and p53-related apoptotic pathway in a high oxidation state. Except for the p53-related apoptotic pathway, mitochondrial PKM2 can interact with Bcl2 and phosphorylates Bcl2 at T69, thus sustaining Bcl2 protein stability and controlling mitochondrial membrane permeability. Besides, PKM2 can also enhance the stability of NF-*κ*B p65 subunit, promoting the binding of NF-*κ*B p65 subunit to Bcl-xL promoter, thereby up-regulating the expression of Bcl-xL protein. PKM2 exerts its effects on apoptosis via the caspases-dependent pathway, including the expression of cleaved caspase 3, caspase 7, and caspase 9.

PKM2 plays a crucial role in cell apoptosis progression [74, 75] (Figure 3). The silencing of PKM2 expression with shRNA or microRNA promotes cell apoptosis in multiple cancer cells [76–79]; the phosphorylation of PKM2 by the dysregulation of microRNAs (miR) has also been reported to regulate cell apoptosis [80–82].

A possible mechanism underlying the effect of PKM2 on mitochondrial apoptosis is that the metabolic function of PKM2 is involved in modulating mitochondrial apoptosis of cancer cells [66, 83, 84]; the enhanced glycolysis by PKM2 can attenuate cell apoptosis in cancer cells [85–87]. Several studies have proven that HIF-1*α*/PKM2 pathway-associated metabolic changes may facilitate apoptosis resistance in cancer cells [88, 89]. The use of metabolic regulation by PKM2 to interfere with cell apoptosis is a new strategy for cancer treatment [90]. PKM2 can also regulate apoptosis via modulation of mitochondrial dynamics. Wu et al. [60] have reported that PKM2-mediated mitochondrial dynamic disorders participate in cell apoptosis. Mitochondrial metabolic and dynamics work together to promote the apoptosis resistance in several cells [66, 81].

On the other hand, PKM2 regulates mitochondrial proteins that are involved in cell apoptosis. PKM2 translocates to the outer membrane of mitochondria under oxidative stress [10]. In the mitochondria, tetrameric PKM2 suppresses the p53 transcriptional activity and p53-related apoptotic pathway in a high oxidation state [78, 91, 92]. Except for the p53-related apoptotic pathway, mitochondrial PKM2 can interact with Bcl2 and phosphorylates Bcl2 at T69, thus sustaining Bcl2 protein stability and controlling mitochondrial membrane permeability [89, 93, 94]. Besides, PKM2 can also enhance the stability of NF-*κ*B p65 subunit, promoting the binding of NF-*κ*B p65 subunit to Bcl-xL promoter, thereby up-regulating the expression of Bcl-xL protein (an anti-apoptotic member of Bcl-2 protein family) [95]. Some studies have reported that PKM2 exerts its effects on apoptosis via the caspase-dependent pathway, including the expression of cleaved caspase 3, caspase 7, and caspase 9 [96–101]. Meanwhile, some other studies have reported that the nuclear translocation of PKM2 is responsible for regulating cell apoptosis, which is caspase-independent, isoform-specific, and independent of its enzymatic activity [102–104].

**Figure 4:**
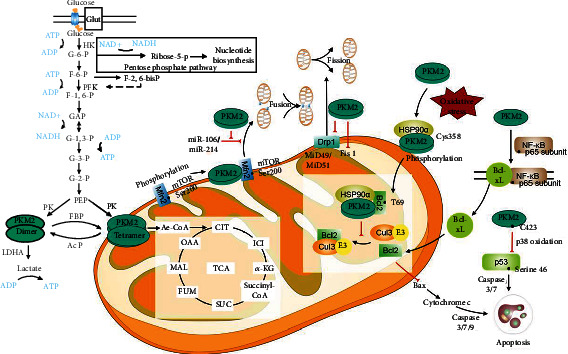
Signaling pathways related to the roles of PKM2 in the regulation of mitochondrial functions.

In summary, this review concerns the related mechanisms of the role of PKM2 in the regulation of mitochondrial functions from the aspects of metabolism, reactive oxygen species (ROS), dynamic, and apoptosis (Figure 4). They may be potentially used in diagnosis and as indicators of disease progression. These findings have increased our understanding of the signaling pathways of PKM2-related mitochondrial functions and indicated that PKM2 may serve as a potential therapeutic intervention for cardiovascular and metabolic diseases [9, 105, 106]. Therefore, the role of PKM2 in mitochondrial functions can be highlighted as a target for the clinical management of cardiovascular and metabolic diseases [17, 20, 107].

## 5. Conclusions

PKM2, known as a key rate-limiting enzyme in glycolysis, is widely involved in the regulation of mitochondrial function, including mitochondrial respiration, reducing ROS damage to mitochondria, mitochondrial morphology, and mitochondrial-dependent apoptosis (Figure 4). Mitochondrial dysfunction plays an important role in the progress of several diseases, especially in cancers. PKM2 can be a potential target for therapeutic intervention in these diseases. However, the current research on the relationship between PKM2 and mitochondrial function is not enough; the specific mechanism of the relationship remains unclear. Further deciphering the functions of PKM2 on mitochondrial function might lead to successful mitochondria-related disease prevention and therapy.

## Data Availability

The data used to support the findings of this study are available from the corresponding author upon request.
